# A Rare Case of Adult Rhabdomyosarcoma

**DOI:** 10.12659/PJR.901967

**Published:** 2017-07-22

**Authors:** Benjamin Sparreboom, Brendan Litton, Julian Yaxley

**Affiliations:** 1 Department of Medical Imaging, The Tweed Hospital, Tweed Heads, NSW, Australia; 2 Department of Medicine, Townsville Hospital, Townsville, QLD, Australia

**Keywords:** Back Pain, Magnetic Resonance Imaging, Rhabdomyosarcoma, Urinary Retention

## Abstract

**Background:**

Rhabdomyosarcoma is a rare, soft tissue malignancy, diagnosed particularly in adults. It commonly metastasizes to the bone marrow. We present a rare case of an adult rhabdomyosarcoma which illustrates the importance of magnetic resonance imaging in identifying early changes in the internal bone structure.

**Case Report:**

A 50-year-old male presented with acute urinary retention. The patient initially had a CT scan of the lumbar spine which only revealed a protrusion of the L5–S1 intervertebral disc and no apparent cause for the patient’s symptoms. One week later, an MRI was performed which showed extensive bone marrow metastases throughout the lumbar spine and a soft tissue mass in the lower sacral region. The bony metastases were not evident on the CT scan and the soft tissue mass was out of the coverage area of the CT. Subsequent biopsy of the soft tissue mass was performed and histopathology concluded the tissue to be a rhabdomyosarcoma. Unfortunately, the patient died one week after diagnosis.

**Conclusions:**

Although adult rhabdomyosarcomas are very rare, this case highlights the advantage of MRI over CT in identifying early changes in the internal bone structure. Therefore, CT should not be relied upon to exclude bony metastases, particularly in the setting of primary cancer with a known tendency to metastasize to the bone marrow.

## Background

Rhabdomyosarcoma (RMS) is a rare, soft tissue malignancy. The aetiopathogenesis of RMS is uncertain, although it is thought to develop from immature cells of the skeletal muscle lineage [1]. RMS is typically found in children and the disease in adulthood is very uncommon [2]. The presenting symptoms and signs of RMS are varied and largely depend on the site of origin of the primary tumour, patient age and the presence or absence of metastases [3]. We report a unique case of a 50-year-old man presenting with urinary retention caused by metastatic RMS with bone marrow and lung metastases detected at the time of diagnosis. Bony lesions were not evident on the patient’s original computed tomography (CT) but were subsequently identified on magnetic resonance imaging (MRI) of the spine, emphasising the importance of MRI in identifying bone marrow metastases.

## Case Report

A 50-year-old male presented to the emergency department of a regional hospital with acute urinary retention. Two weeks earlier, he had been commenced on paracetamol and codeine for back pain and left leg sciatica which were troublesome since moving a refrigerator 3 weeks earlier. The patient’s medical history was notable for a renal transplant at 20 years of age, performed for end-stage renal failure secondary to childhood glomerulonephritis. He was also positive for factor V Leiden mutation and suffered from deep vein thrombosis 18 months earlier. Neurological examination was non-contributory. His urinary retention was initially attributed to opiate overuse and constipation and was relieved by temporary catheter drainage. Opiates were ceased and the patient was discharged from the emergency department.

The patient returned the following day with further urinary retention. An indwelling urinary catheter was placed and the patient was discharged and asked to return to perform a trial-of-void 3 days later. The trial-of-void was subsequently unsuccessful and given his ongoing back pain, CT of the lumbar spine was performed, demonstrating a protrusion of the L5–S1 intervertebral disc; however, it did not provide a conclusive cause for the patient’s urinary retention. He was observed overnight in the emergency department and again discharged the following day with a urinary catheter *in situ*.

MRI of the lumbar spine was arranged the following week. It revealed extensive bony metastatic disease throughout the lumbar spine with associated soft tissue masses at the level of the lower sacrum extending into the sciatic notch. The bony metastases were not evident on the initial lumbar spine CT and the soft tissue masses were not within the coverage of the CT. On MRI, the vertebral lesions demonstrated low T1 and T2 signals, and a high STIR signal.

The patient was admitted to hospital and CT-guided biopsy of the low sacral soft tissue mass was performed. Histopathology concluded that the tissue was RMS with pleomorphic histology, the most common histological subtype encountered in adult RMS [4]. Staging scans performed at this time revealed widespread pulmonary metastases but no convincing thoracic, abdominal or pelvic primary mass.

**Figure 1:**
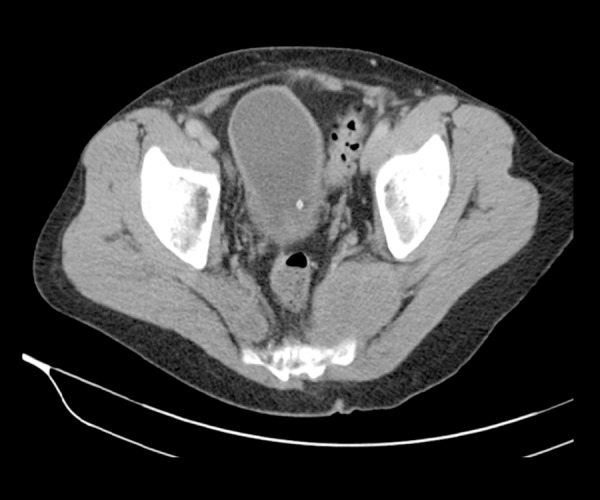
Image of the biopsied mass on CT in axial plane.

**Figure 2:**
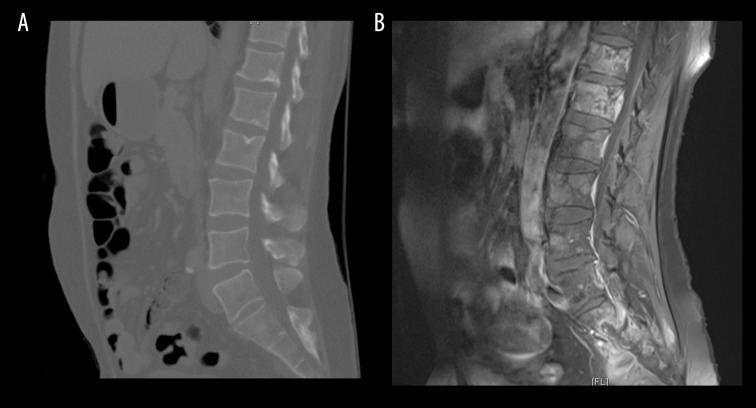
(**A**) CT lumbar spine image in sagittal section showing no evidence of metastatic disease. (**B**) T1, fat-saturated image in sagittal section showing extensive bony metastatic disease on MRI performed only one week after the CT of lumbar spine.

The patient had previously had an MRI of a longstanding, right anterior leg mass performed several weeks prior to his initial presentation to hospital. The mass had been present for approximately 12 months. MRI findings were at that time thought to suggest a nerve sheath tumour. No tissue diagnosis was available on this mass but it was hypothesised to represent another RMS focus. The patient started palliative radiotherapy to the sacral mass and one of the pulmonary masses. Systemic chemotherapy was planned; however, the patient’s symptoms rapidly progressed and he died only one week after diagnosis (Figures 1, 2).

## Discussion

RMS is a rare, soft tissue malignancy of a mesenchymal origin, suspected to arise from cells of the skeletal muscle lineage [1]. RMS is very uncommon in adults and constitutes only 3% of all soft tissue sarcomas [3]. The commonest site of metastasis is the lungs followed by bone marrow and lymph nodes [3], as demonstrated in our case. Most primary adult RMSs occur in the extremities, and the lower limb mass in our patient was highly suspicious of the primary tumour. Moreover, adult RMS is extremely aggressive with 5-year survival rates of approximately 27%, which is lower than survival rates in paediatric populations [3]. Acute urinary retention can be one of the first signs of rhabdomyosarcoma of the bladder or prostate [4]. However, in our case there was no radiologic evidence of urinary tract involvement and we postulate that the symptoms of urinary retention and sciatica were a manifestation of sacral nerve root compression by the surrounding mass.

Following arrival at a histological diagnosis, staging involves a full history, physical examination and routine laboratory tests. Evaluation at and around the primary site is typically made with CT and/or MRI and abdominal ultrasound [5]. However, for evaluation of tumours in pelvic and limb sites two studies recommend MRI over CT, except where cortical bone involvement may influence the management [3,6].

On contrast-enhanced CT, pleomorphic RMS appears isodense-to-hyperdense compared to skeletal muscles. On T1-weighted MRI, pleomorphic RMS tends to appear isointense-to-hyperintense compared to the skeletal muscle with variable contrast. Pleomorphic RMS typically has high signals on T2-weighted/Short Tau Inversion Recovery (STIR) imaging with extensive heterogeneity [3]. These features correlate with the imaging findings observed in our case. Additionally, the soft tissue masses in this case showed mild, predominantly peripheral, post-contrast enhancement.

## Conclusions

It has been shown that MRI reveals early bone marrow metastases before any changes in the internal bone structure become evident on CT [6]. This has been exemplified in our case and highlights the importance of MRI and its superiority over CT in identifying replacement of normal bone marrow. On this basis, CT should not be relied upon to exclude bony metastases, particularly in the setting of primary cancer with a known tendency to metastasise to the bone marrow.
